# Kidney Stones, Proteinuria and Renal Tubular Metabolic Acidosis: What Is the Link?

**DOI:** 10.3390/healthcare10050836

**Published:** 2022-05-02

**Authors:** Maxime Ilzkovitz, Elikyah Esther Kayembe, Caroline Geers, Agnieszka Pozdzik

**Affiliations:** 1 Kidney Stone Clinic, Nephrology Department, Hôpital Brugmann, 1020 Brussels, Belgium; elikyah.kayembe@stpierre-bru.be; 2 Pathology Department, Hôpital Brugmann, 1020 Brussels, Belgium; caroline.geers@chu-brugmann.be; 3 Faculty of Medicine, Université Libre de Bruxelles (ULB), 1050 Brussels, Belgium

**Keywords:** renal tubular acidosis, nephrocalcinosis, Sjögren syndrome, chronic kidney disease, hypocitraturia

## Abstract

Kidney stone disease represents a rare cause of chronic kidney disease (2–3%) but has severe clinical consequences. Type 1 renal tubular acidosis is a strong lithogenic condition mainly related to primary Sjögren syndrome. This study aimed to illustrate an unusual presentation of Sjögren syndrome to improve the knowledge about rare kidney stone diseases, and to provide clues for the diagnostic approach in this specific condition. We report the case of a 35-year-old Indian woman with severe nephrocalcinosis and chronic kidney disease with tubular proteinuria who presented for metabolic assessment. We found advanced chronic kidney disease, low serum bicarbonate, permanent alkaline urine with pH at ~7.1, and severe hypocitraturia corresponding to type 1 renal tubular acidosis. The erythrocyte sedimentation rate was high. Serological screening for HAV, HBV, HCV, HIV, EBV was negative and complement was normal. Autoimmune screening showed antinuclear antibodies (>1/1.280) with anti-SSA, anti-SSA/Ro52 and anti-SSB antibodies. Genetic testing excluded an inherited cause of renal tubular acidosis. A renal biopsy showed moderate chronic tubulo-interstitial nephritis without any glomerular involvement. Primary Sjögren syndrome with significant renal involvement was considered, and corticosteroids were then subsequently initiated in combination with potassium citrate with vitamin D substitution. Only partial improvement was observed in electrolytes disturbance. After 15 months, her renal function remained stable. In conclusion, nephrocalcinosis could be the first manifestation of severely impacting diseases such as primary Sjögren syndrome. Chronic kidney disease, bilateral nephrocalcinosis, and metabolic acidosis can be linked through type 1 renal tubular acidosis. Therefore, autoimmune screening for Sjögren syndrome should be considered in such cases.

## 1. Introduction

Diabetes mellitus and systemic arterial hypertension are the most common etiologies of chronic kidney disease. They are followed by glomerulonephritis, herbal or environmentally induced nephropathies and kidney diseases related to HIV, HBV and HCV infections [1]. Nephrolithiasis and nephrocalcinosis are underestimated causes of chronic kidney disease and end-stage renal disease [2,3]. The estimated prevalence of kidney stone diseases ranges from 1 to 13% with an incidence that almost tripled between 1976–2010 in the United States (from 3.2 to 8.8%) [4,5]. Kidney stone diseases are rarely the primary cause of chronic kidney disease (2–3%) but have severe consequences when this is the case [6,7]. In fact, end-stage renal disease is from two to three times more common in this specific population, independent of cardiovascular comorbidities [8,9]. Decline in renal function in kidney stone disease may result from obstructive uropathy, secondary pyelonephritis or their treatments, anion accumulation-induced inflammation or from an underlying disease [6,10].

Kidney stone diseases are usually classified according to their stone morpho-constitutional analysis. Metabolic workup identifies the current urinary abnormalities that need to be corrected to reduce the risk of stone recurrence.

Type 1 renal tubular acidosis is recognized as one of the most lithogenic diseases [5,11] characterized by pathognomonic type IVa2 stone morphology. Inherited type 1 renal tubular acidosis conditions are rare. Mutations are located on genes coding for H+(V)/ATPase (ATP6V1B1 and ATP6V0A4) or AE1 anion exchanger (SLC4A1). Acquired type 1 renal tubular acidosis are more frequent and result from renal tubular damage due to autoimmune diseases, metabolic diseases, or drugs [12].

Acidemia results from a defect in H+(V)/ATPase (apical excretion of H+ which binds to ammonia to form urinary ammonium) or in AE1 anion exchanger (basal re-uptake of bicarbonate against chloride ion). This causes a renal acidification defect by the blood excess of acid charge. It is first balanced by the bicarbonate buffer and then the bone buffer responsible for bone resorption leads to secondary osteopenia and osteoporosis. Released phosphates are filtrated and bind urinary proton (H+) inducing an increase in urine pH and a decrease in citraturia. Finally, calcium–phosphate supersaturation is promoted by alkaline urine, hypocitraturia, and hypercalciuria [12]. Histologically, calcium–phosphate stone plugs are found in inner medullary collecting ducts and ducts of Bellini associated with tubulointerstitial inflammation, interstitial fibrosis, tubular atrophy and ischemia [6].

The aim of this study was to illustrate the unusual presentation of a renal Sjögren disease, to improve knowledge about rare kidney stone disease and to provide clues for that diagnosis.

## 2. Case Description

A 35-year-old woman from India who was living in Belgium for 6 years was referred for kidney stone disease and chronic kidney disease. She reports kidney stone disease for 15 years with moderate activity (one episode every five years) and chronic kidney disease since 2017. She has hypothyroidism and hypertriglyceridemia. There is no familial history of chronic kidney disease nor kidney stone disease. She did not report a history of alcohol or drug abuse. Her current medication consists of L-thyroxine (100 µg daily).

At the admission, the patient had no complaint, no hearing loss or ocular disturbance (sicca syndrome absent). Vital parameters and the physical exam were unremarkable (weight: 55.6 kg, BMI: 23.4 kg/m^2^, blood pressure: 113/70 mmHg, heart rate: 73/min, body temperature: 35.9 °C).

The blood tests demonstrated: hemoglobin: 13.8 (N 12.0 to 16.0 g/dL); eosinophilia: 0.54 (N < 0.40 × 10^3^/μL); urea: 34 and creatinine (Cr): 1.48 (N 17–48 and 0.50–0.90 mg/dL, respectively); GFR (CKD-EPI): 46 mL/min/1.73 m^2^; bicarbonate: 16 (N 23–29 mmol/L); chlore: 107 (N 98–107 mmol/L); potassium: 3.0 (N 3.4–4.4 mmol/L); total calcium: 2.39 (N 2.20–2.70 mmol/L); ionized calcium 1.30 (N 1.05–1.30 mmol/L); phosphorus: 0.93 (N 0.75–1.39 mmol/L); anion gap: 14 (9–21 mEq/L); alkaline phosphatase 78 (N 30–120 UI/L); calcidiol: 19.2 (N 30.0–80.0 µg/L); bioactive parathyroid hormone level: <10 (N < 49 ng/L); thyrotropin level: 0.87 (N 0.27–4.20 mU/L); free thyroxine level: 14.1 (N 12.0–22.0 pmol/L) with anti-TPO 270 (N < 24 kUI/L) and normal values for other liver enzymes and bilirubin.

The C-reactive protein level was normal, but an erythrocyte sedimentation rate was at 64 (N < 20 mm/h). The serum protein electrophoresis showed polyclonal gammaglobulinemia at 24.2 (N 8.0–13.5 g/L). Autoimmunity screening was positive for rheumatoid factor 52.6 (N < 14.0 UI/mL) and antinuclear antibodies (>1/1280) with a fine-granular nuclear pattern corresponding to an anti-SSA (high titer), anti-SSA Ro52 (high titer), anti-SSB (mild titer) and anti-histon (moderate titer) antibodies. Anti-dsDNA, anti-Sm, anti-nucleosome antibodies are negative. The lupus anticoagulant (dRVVT test) was positive. Anti-neutrophil cytoplasmic antibodies, anti-glomerular basement membrane antibodies and cryoglobulinemia were negative. The C3 and C4 complement components are within the normal range. Serological screening for HAV, HBV, HCV, HIV, and EBV was negative.

**Figure A1:**
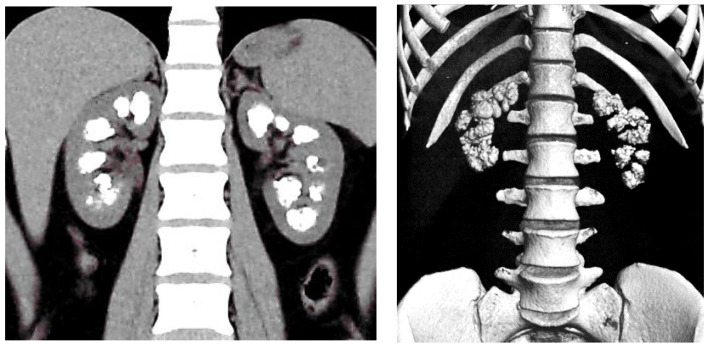
Coronal reformation of unenhanced CT-scan shows bilateral numerous pre-calyceal lithiasis and severe medullary nephrocalcinosis but without uretero-hydronephrosis.

The urine dipstick shows the pH at 7.0, traces of proteins, hematuria without acanthocytes (RBC: 48/muL), and leukocyturia (WBC: 98/muL) without crystals. The urine was sterile. The fasting urinary spot showed proteinuria at 1.0 g/Cr, albuminuria 150 mg/gCr (tubular, non-selective proteinuria) without glycosuria and a positive urinary anion gap (29 mmol/L). The urinary metabolic work-up reported a urine volume of: 2150 mL/24 h, uricuria: 327 mg/24 h, natriuresis: 100 mmol/24 h, calciuria: 2.5 mmol/24 h (1.14 mmol/L), oxaluria: 27.4 mg/24 h and severe hypocitraturia: <108 µmol/24 h. Weight-based urinary calcium was 1.8 mg/kg/day. The fasting urine test showed a pH of 7.1, urine density at 1.008, and calciuria at 1.33 mmol/L (<3.5 mmol/L). Kidney stones could not be assessed by morpho-constitutional method and no urinary crystals were detected. Renal ultrasound showed bilateral macroscopic kidney stones with calyceal and ureteral dilatation and abdominal CT scan confirms bilateral numerous pre-calyceal lithiasis and severe medullary nephrocalcinosis without uretero-hydronephrosis (Figure A1). Diagnostic ureterorenoscopy was performed and demonstrated calyceal but mainly submucosal stones.

**Figure A2:**
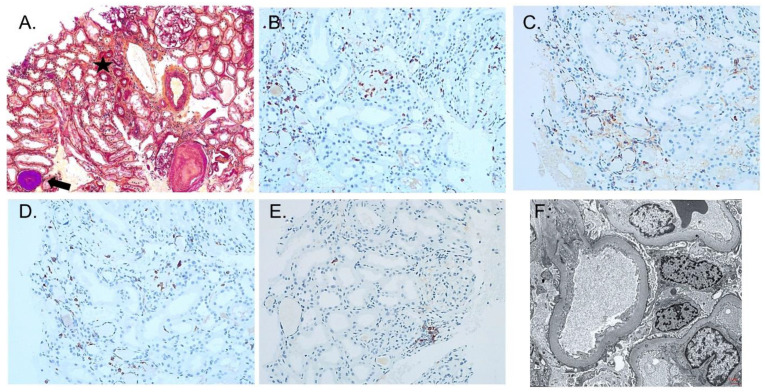
Kidney biopsy. Renal biopsy tissue was fixed in paraffin and sections were stained with hematoxylin and eosin. Immunophenotyping was performed by immunostaining (CD3, CD4, CD8, CD20).

To determine the type of primary nephropathy considering the proteinuria, we performed a renal biopsy consistent with moderate acute and chronic tubulointerstitial nephritis without glomerular disease (absence of IgA, IgM, IgG, C3, C1q, kappa, and lambda light chains immunofluorescence) (Figure A2). Von Kossa staining was negative. Inherited type 1 renal tubular acidosis was excluded as the genetic screening for the mutation of tubulopathies including genes coding for H+(V)/ATPase (ATP6V1B1 and ATP6V0A4) or AE1 anion exchanger (SLC4A1) was negative.

As we considered the diagnosis of the primary Sjögren syndrome with type 1 distal renal tubular acidosis associated with chronic tubulointerstitial nephritis, we introduced potassium citrate (6 g/day) with a slight improvement on ions disturbance and we associated 0.5 mg/kg prednisolone equivalent therapy. After 15 months since the beginning of treatment, she is doing well. She has remained free of acute attacks.

## 3. Discussion

We report a case of primary Sjögren syndrome with renal involvement presenting as complete type 1 renal tubular acidosis, chronic tubulointerstitial nephritis, nephrolithiasis, and nephrocalcinosis leading to advanced chronic kidney disease. We will further discuss the relationship between these specific clinical entities subsequently.

The chronic kidney disease stage 3a is associated with mixed tubular range proteinuria, microscopic hematuria, leukocyturia, and severe nephrocalcinosis without hypertension. Her autoimmune profile (high titer of antinuclear antibodies associated with anti-SSA/SSB antibodies, absence of anti-dsDNA and anti-Sm antibodies) suggested primary Sjögren syndrome and was supported by histological findings (diffuse tubulointerstitial nephritis with predominant T-cell infiltrate in the absence of glomerular disease) despite not reaching classification criteria, and especially no sicca syndrome [13,14]. Tubulointerstitial nephritis is the main presentation of renal involvement in primary Sjögren syndrome. Underdiagnosed, type 1 renal tubular acidosis and tubulointerstitial nephritis are reported to occur 2–7 years after the onset of primary Sjögren syndrome [15] and can already develop before sicca symptoms [16]. Moreover, an isolated lupus anticoagulant was also reported in primary Sjögren syndrome [17].

Renal involvement in primary Sjögren syndrome ranges from 0.5 to 15% in Europe and the United States of America to more than 30% in Asian populations (1). The clinical features related to kidney involvement encompassed a broad-spectrum including electrolytes disturbances, tubulointerstitial nephritis (75%), nephrolithiasis (9.5%) and nephrocalcinosis (5.3%) (2) or chronic kidney disease in the absence of an early diagnosis and adequate treatment. Renal involvement was positively with anti-SSB and negatively associated with arthralgia (3).

The moderate activity of the disease contrasts with the massive impact of nephrocalcinosis on renal function. Calcium phosphate-based stone disease is the most likely diagnosis in this case. An underestimation of calciuria in the context of chronic kidney disease and vitamin D deficiency may explain the absence of hypercalciuria in our patient. Alkaline urinary pH, low urinary oxalate and low urinary citrate are the other lithogenic factors needed for calcium phosphate crystals precipitation. Urinary citrate is a natural calcium chelator which reduces calcium oxalate supersaturation. Urinary citrate is reduced by hypokalemia and alkaline urine but lower levels of calciuria have also been described in stone formers with chronic kidney disease [18]. In our case, nephrocalcinosis is related to severe secondary type 1 renal tubular acidosis. Indeed, autosomal recessive mutations of H+(V)/ATPase (ATP6V1B1 and ATP6V0A4) were unlikely because these generally present with hearing loss and growth retardation during childhood. However, the autosomal dominant mutation of AE1 anion exchanger (SLC4A1) was more plausible as it may have late-onset expression without hearing loss [19]. We excluded the involvement of both autosomal recessives and autosomal dominant mutation in type 1 renal tubular acidosis.

The lack of supporting history along with normal liver tests do little to support drug use problems, metabolic disease (e.g., Wilson disease), auto-immune hepatitis, and primary biliary cholangitis. Indeed, primary Sjögren syndrome is likely the most frequent cause of acquired hypokalemic distal renal tubular acidosis [20]. Tubular defects associated with LES are scarce and usually associated with hyperkalemic renal tubular acidosis (type 4) [21]. Sarcoidosis remains an exclusion diagnosis. Medullary sponge kidney disease remains challenging to exclude since the disease is very severe.

In this study, we report details on the cells infiltrating the tubulointerstitial compartment in primary Sjögren syndrome. Following the literature, our case demonstrates a T-cell predominant infiltrate. Jasiek et al. reported that tubulointerstitial infiltrate in primary Sjögren syndrome is T-cell predominant in 65%, plasma-cell predominant in 25%, and B-cell predominant in 10% of cases [22].

This case highlights the importance of performing a renal biopsy when autoimmune-associated chronic kidney disease is suspected. It is essential to exclude a glomerular disease but also to document interstitial inflammation. In primary Sjögren syndrome, tubulointerstitial nephritis impacts prognosis and its early detection improves response to treatment. A biopsy should be performed once renal function decreases to guide treatment [15].

The treatment of renal primary Sjögren syndrome is poorly established but relies on ESSDAI [5]. The treatment of type 1 renal tubular acidosis consists of the correction of acidosis with potassium citrate to prevent nephrolithiasis and to reduce morbi-mortality in the case of chronic kidney disease [20,23]. If tubulointerstitial nephritis is associated with an autoimmune disease, a high corticosteroid regimen (>0.5 mg/kg prednisolone equivalent) is recommended to stabilize eGFR. The success of corticoids depends on the rate of chronic interstitial fibrosis [15,24].

Mycophenolate mofetil shows promising results but randomized controlled studies are still needed to determine whether immunosuppressive therapy can improve renal prognosis compared to corticosteroids [25].

Association between primary Sjögren syndrome and thyroid diseases is not well elucidated. Whether there is a common pathophysiologic pathway or both diseases predominantly occur in the same population is not clear. Meta-analysis demonstrates that thyroid diseases are three times more frequent in patients with Sjögren syndrome, particularly autoimmune hypothyroidism. Patients with concomitant Sjögren syndrome and autoimmune thyroid diseases have a stronger chance of developing other autoimmune diseases and lymphoproliferation [26]. Hypothyroidism treatment with hormone substitution can promote calcium-based urolithiasis by osteoclasts stimulation and therefore also hypercalcemia and hypercalciuria [27].

## 4. Conclusions

In patients with urolithiasis, hypocitraturia, and anti-SSA/SSB, Sjögren syndrome diagnosis should be considered. Lithogenic factors in type 1 renal tubular acidosis are alkaline urine and low citraturia. Renal involvement could be the first clinical manifestation of primary Sjögren syndrome and varies from ionic disturbances, tubulointerstitial nephritis or nephrolithiasis, to nephrocalcinosis resulting in chronic kidney disease in the absence of early diagnosis and adequate treatment. Randomized controlled studies are needed to determine the role of immunosuppressive therapy.

## Data Availability

Not applicable.

## Appendix A

**A**. Microscopic evaluation shows 12 glomeruli, among which 4 are sclerotic. Glomeruli showed discrete diffuse mesangial thickening, slightly increased mesangial cellularity, the absence of inflammatory infiltration or necrosis. Bowman’s capsule was slightly thickened and fibrous. There were no signs of arteriosclerosis and the arterioles were normal. Within the tubulointerstitial compartment we found mild polymorph tubulointerstitial inflammation (*) (lymphocytes, plasma cell, and a few eosinophils) without granuloma, and diffuse interstitial fibrosis (less than 50%) with tubules injuries. Several groups of tubules were atrophied with a thickened tubular basement membrane and lumens filled with proteinaceous material (→). Some tubes were dilated by very thick proteinaceous (H & E ×100). Representative images of: **B**: anti-CD3; **C**: anti CD4; **D**: anti CD8; and **E**: anti-CD 20 immunostainings (H & E ×300). **F:** Electron microscopy examination shows a segment of normal glomerulus. The capillary wall had preserved architecture. The endothelial cells did not show any abnormalities. The glomerular basement membrane was thin and regular with few slightly thickened and fibrous areas. The podocytes showed normal pedicels. There were no deposits in the mesangium or in the basement membrane. Original magnification: 33.000. In conclusion, renal parenchyma presented with chronic T-cell predominant tubulointerstitial nephritis (mild chronic damage, with moderate interstitial fibrosis, mild glomerulosclerosis, and mild tubular atrophy).
